# A genetic association study between growth differentiation factor 5 (*GDF 5*) polymorphism and knee osteoarthritis in Thai population

**DOI:** 10.1186/1749-799X-6-47

**Published:** 2011-09-21

**Authors:** Tulyapruek Tawonsawatruk, Theeraroj Changthong, Sarinee Pingsuthiwong, Objoon Trachoo, Thanyachai Sura, Wiwat Wajanavisit

**Affiliations:** 1 Department of Orthopedics, Faculty of Medicine, Ramathibodi Hospital, Mahidol University, Bangkok 10400, Thailand; 2 Division of Medical Genetics and Molecular Medicine, Department of Medicine, Faculty of Medicine, Ramathibodi Hospital, Mahidol University, Bangkok 10400, Thailand

**Keywords:** Osteoarthritis, *Growth Differentiation Factor 5*, *GDF5*, SNP, RFLP, Thais

## Abstract

**Objective:**

Osteoarthritis (OA) is a multi-factorial disease and genetic factor is one of the important etiologic risk factors. Various genetic polymorphisms have been elucidated that they might be associated with OA. Recently, several studies have shown an association between *Growth Differentiation Factor 5*(*GDF5*) polymorphism and knee OA. However, the role of genetic predisposing factor in each ethnic group cannot be replicated to all, with conflicting data in the literatures. Therefore, the aim of this study was to investigate the association between *GDF5 *polymorphism and knee OA in Thai population.

**Materials and Methods:**

One hundred and ninety three patients aged 54-88 years who attended Ramathibodi Hospital were enrolled. Ninety cases with knee OA according to American College of Rheumatology criteria and one hundred and three cases in control group gave informed consent. Blood sample (5 ml) were collected for identification of *GDF5 *(*rs143383*) single nucleotide polymorphism by PCR/RFLP according to a standard protocol. This study protocol was approved by the Ethics Committee on human experimentation of Ramathibodi Hospital Faculty of Medicine, Mahidol University. Odds ratios (OR) and 95% confidence intervals were calculated for the risk of knee OA by genotype (TT, TC and CC) and allele (T/C) analyses.

**Results:**

The baseline characteristics between two groups including job, smoking and activity were not different, except age and BMI. The entire cases and controls were in Hardy-Weinberg equilibrium (p > 0.05). The OA knee group (n = 90) had genotypic figure which has shown by TT 42.2% (n = 38), TC 45.6% (n = 41) and CC 12% (n = 11), whereas the control group (n = 103) revealed TT 32% (n = 33), TC 45.6% (n = 47), and CC 22.3% (n = 23), respectively. Genotypic TT increased risk of knee OA as compared to CC [OR = 2.41 (P = 0.04, 95%CI = 1.02-5.67)]. In the allele analysis, the T allele was found to be significantly associated with knee OA [OR = 1.53 (P = 0.043, 95%CI = 1.01-2.30)].

**Conclusion:**

These data suggested that *GDF5 *polymorphism has an association with knee OA in Thai ethnic. This finding also supports the hypothesis that OA has an important genetic component in its etiology, and *GDF5 *protein might play important role in the pathophysiology of the disease.

## Introduction

It is widely believed that osteoarthritis develops from an imbalance between anabolic and catabolic processes or homeostasis of cartilage metabolism [1,2]. The etiology of this disease is related to genetic association [3]. Recently, several studies have demonstrated the polymorphism in many genes which might be related to the pathogenesis of osteoarthritis [4-14]. *Growth Differentiation Factor 5 *or *GDF5 *gene regulates the expression of the *GDF5 *protein which is closely related to BMP and is a member of TGF-beta superfamily [15]. It has a role in regulation of the chondrogenesis and the defect in this gene might be correlated to the abnormal joint development [15]. It has been reported in animal study that *GDF5 *knockout mice develops knee joint anomaly [16]. Moreover, it has been shown that he polymorphism in *GDF5 *gene is related with low expression of the *GDF5 *protein in knee joint [17].

The large scale analysis has shown that the association of this polymorphism (*rs143383*) in promoter area might be a risk factor of the osteoarthritis [18]. In addition, studies of this genetic variant in China and Japan have shown the association of T allele and knee OA [19]. However, there is a report from Greece which had an inconsistent result and found no an association [20]. The genetic susceptibility of the disease in different ethnic cannot be applied to others because each ethnic has a different genetic background. There is a gap of information about this polymorphism in Thai population, therefore the objective of this study is to determine the association of the SNP *rs143383 *in *GDF5*gene and knee OA in Thai population.

## Patients and Methods

### Subjects

Our study was approved by the Ethics Committee of the Faculty of Medicine, Ramathibodi Hospital, Mahidol University, Bangkok, Thailand. All patients recruited in the study were Thai by nationality and had ancestors settled in Thailand for at least three generations. In total, 90 patients with knee OA who underwent total knee arthroplasty (TKA) and 103 patients without knee OA were enrolled from Department of Orthopedics, Ramathibodi Hospital. Informed consent was performed after the purpose of the research project and it had been clearly explained to the patients. The diagnosis of knee OA was based on the American College of Rheumatology criteria [21]. Both OA and control groups were interviewed to obtain demographic data and all of established risk factors. Thereafter, standard weight-bearing antero-posterior and lateral view of knee radiographs were taken to confirm the diagnosis of OA by Kellgren and Lawrence scores (KL scores) [22].

### Laboratory technique

#### PCR-RFLP for BsiEI restriction site was used for SNP in GDF-5 identification

**Figure 1:**
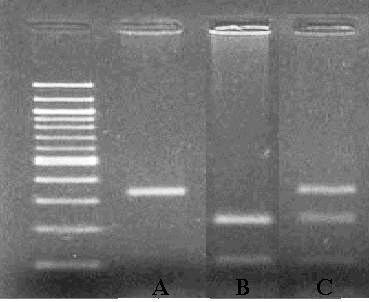
**shows the bands of each genotype in the electrophoresis gel: A represents TT, B represents CC and C represents TC**.

Firstly, 5 ml peripheral blood sample was collected from patient using ethylenediamine tetraacetic acid as an anticoagulant and processed for SNP analysis. Genomic DNA was extracted from buffy coat leukocytes using the standard phenol-chloroform method. PCR primers to amplify the promoter area of GDF5 gene were designed by the Primer-3 web-based tool [23]: GATTTTTTCTGAGCACCTGCAGG (forward) and GTGTGTGTTTGTATCCAG (reverse). 50 μl PCR mixture contained 100 ng of genomic DNA, 20 pmol of each primer, 0.2 μ M of each dNTP, 1 unit of Taq DNA polymerase (AmpliTaq^®^, Applied Biosystem, Foster City, CA), 3.0 mM MgCl2 in 10 × PCR buffer containing 10 mmol of Tri-HCl pH 9.0, 10 mmol KCl and 0.1% Triton X-100 (Invitrogen, Carlsbad, CA). PCR reaction was started with an initial denaturation at 95°C for 5 min, followed by 35 cycles of amplification in a thermocycler (PCR Sprint, Thermofisher, Waltham, MA) with denaturation at 94°C for 1 min, annealing at 58°C for 1 min, extension at 72°C for 1 min, and final extension at 72°C for 10 min. Then, 10 μL of PCR product was incubated at 37°C with 3 units of BsiEI for 4 hours under the manufacturer's recommended conditions (New England Biolabs, Ipswich, MA). The digested product was electrophoresed on 2% agarose gel with ethidium bromide staining before being visualized on a UV transilluminator (Figure 1). The expected fragment length was 104 and 230 bp in CC, 104, 230, and 344 bp in TC, and 344 bp in TT genotypes, respectively.

### Statistical analyses

Analysis of demographic data was performed by Excel 2007 (*Microsoft*^® ^*Excel*^®^). The unpaired t-test was used for continuous data and Chi-square was used for categorical data. Allele frequencies, Odds ratio and the probability for Hardy-Weinberg Equilibrium were estimated and analyzed as explained by following website; http://ihg.gsf.de/cgi-bin/hw/hwa1.pl.

## Results

In our study, the average age of patients in the knee OA group was significant older than in the control. BMI which is one of the risk factor of knee OA was also slightly higher in the OA patient group. However, other possible risk factors which might be related to OA as well as patient life style were comparable between these two groups. The baseline characteristics of the patients are shown in Table 1. The prevalence of allele T and C in our sample was normally distributed according to the Hardy-Weinberg Equilibrium (p-value < 0.05). The comparison of the number and odd ratio of genotype TT, TC, TT+TC with CC of *GDF5 *polymorphism (*rs143383*) in the promoter area between case and control is demonstrated in Table 2. When analyzing by allele (shown in table 3), the odds ratio of T allele was 1.53 (95%C.I. = 1.01-2.31), which delineated the increase in risk to develop knee OA. Our result shown that this polymorphism, T/C, is inherited by autosomal recessive manner as the TT genotype increases risk of the disease significantly (OR = 2.41, 95%C.I. = 1.02-5.67), whereas the TC has no significant difference.

**Table 1:** Shows the base line characteristics between cases and controls

Variables	**Cases **(n = 103)	**Control **(n = 103)	p-value
Age (years), mean (SD)	68.46(10.0)	59.25(9.0)	< 0.01*
Onset of disease (years), mean (SD)	61.46(9.06)	N/A	N/A
Female (%)	79(87.25)	93(90.29)	0.7
BMI (kg/m^2^), mean (SD)	26.66(4.0)	24.57(5.0)	< 0.01*
History of labor work (%)	25(27.78)	30(29.13)	0.20
Regular exercise (%)	10(11.11)	13(12.62)	0.15
Kneeling activity (%)	37(41.11)	48(46.60)	0.07
Previous any fracture (%)	9(10.0)	13(12.62)	0.15
Smoking (%)	2(2.22)	4(3.88)	0.10

Note: *statistically significant, SD = standard deviation, N/A = not applicable

**Table 2:** Shows the association between each genotype and risk of knee OA

Genotype	**Case **(n = 90)	**Control **(n = 103)	**Odds ratio **(95%CI)	p-value
TT	38	33	2.41(1.022-5.670)	0.04*
TC	41	47	1.82(0.794-4.190)	0.15
TT + TC	79	80	2.07(0.944-4.517)	0.06
CC	11	23	1	N/A

(CC was used for calculation Odd ratio of TT, TC and TT+TC)

Notes: *statistically significant, N/A = not applicable

**Table 3:** Shows the association between allele and knee OA

Allele	Case	Control	**Odds ratio **(95%CI)	p-value
**T**	117	113	1.53(1.013-2.306)	0.04*
**C**	63	93	1	N/A

(C was used for calculation Odd ratio of T)

Notes: *statistically significant, N/A = not applicable

## Discussion and Conclusion

Our study has shown the statistically significant association between polymorphism in promoter of *GDF5 *gene (*rs143383*) and knee osteoarthritis in Thai population. We found that T allele in *GDF5 *polymorphism was a significant risk factor for knee osteoarthritis with Odds ratio 1.53 (95%C.I. = 1.01-2.31). When analysed by genotype, it was found that the TT genotype increased risk of knee OA, whereas TC has not shown any correlation. Regarding these finding, it suggests that this polymorphic gene might be expressed as the autosomal recessive type. The *GDF5 *gene is located on the chromosome 20q11.2 and it regulates the expression of *GDF5 *protein. It is categorised in the class of bone morphogenetic protein (BMP) [15]. It involves in development of bone and cartilage, particularly in endochondral ossification process [24,25]. The mutation of *GDF5 *associates with generalised osteoarthritis and skeletal-related congenital diseases. Acromesomelic chondrodysplasia; Grebe type and Hunter-Thompson type is autosomal recessive form which is rare hereditary skeletal disorders. They are presented by short status, abnormal limbs development. Brachydactyly type A1, A2 and C and symphalangism proximal syndrome are inherited malformation presented with abnormal morphology of hand and finger. Multiple synostoses syndrome type 2 is an autosomal dominant condition characterised by progressive joint fusions and progressive conductive deafness. Du Pan syndrome is a rare autosomal recessive; the patient presents with absence of the fibulae and severe acromesomelic limb shortening with small, non-functional toes. These abnormal conditions have been reported that they have a mutation in GDF5 gene, thus they support that GDF5 plays a role in skeletal development [26,27]. Recently, it has been reported that GDF5 deficiency mice had the delay fracture healing process that impaired the cartilaginous matrix deposition in the callus and reduced callus cross-sectional area [28].

The functional study of this polymorphism has been demonstrated; T allele in rs143383 was associated with the decrease of *GFD5 *molecule expression and might increase susceptibility to osteoarthritis [17]. Moreover, it has been report that the differential binding of deformed epidermal autoregulatory factor 1 (DEAF-1) can modulate the expression of *GDF5 *via this polymorphism [29]. It is believed that *GDF5 *plays role in the regulation of the chondrogenic cell growth and differentiation. These evidences support the function of *GDF5 *gene which might indicate the importance of this polymorphism in osteoarthritis etiology. As the result, many scientists have been working for study the association of this polymorphism in *GDF5 *gene. Recently, a large scale association study between *GDF5 *gene and osteoarthritis has revealed the genetic susceptibility of polymorphism in the *GDF5*; *rs143383 *with odds ratio 1.12 (95%CI 0.99-1.31) [18]. This study included the data from diverse ethnics which have been reported in the recent literatures. However, the magnitude of this association is smaller than the Asian ethnic, it has been reported from Japan that odds ratio is 1.79 (P = 1.18 × 10-13) for *GDF5 *polymorphism with per-risk allele (T) odds ratio by Miyamoto et al [17]. The magnitude of the association of *GDF5 *polymorphism is different between Caucasian ethnic and Asian ethnic. Furthermore, the association of this polymorphism from Greek and Korean study cannot demonstrate the association of *GDF5 *polymorphism [20,30]. We have also reported the polymorphism in *ESR1*, which is associated with knee OA in Korean population. However, our result did not show any statistically significant difference [31]. Thus, these evidences can imply that the association of ethic and genetic susceptibility in osteoarthritis might not be consistent among the different populations and cannot be applied to others.

In addition, there is a report that *GDF5 *polymorphism (*rs143383*) also predisposes to Lumbar disc degeneration in women. Lumbar disc degeneration which is defined by disc space narrowing and the presence of osteophytes significantly associates with GDF5 polymorphism from cohorts from Northern Europe in women, with an odds ratio (OR) of 1.72 (95% CI 1.15-2.57) [32]. The genome-wide association study from Finland and Sardinia shows the common variants in GDF5 contribute to height difference [33]. The GDF5 gene might be involved skeletal growth and development and GDF5 variants might play role in pathogenesis of bone and cartilage diseases.

Although the sample size in our study was small when compared to previous studies, these limited samples were sufficient for demonstrating the statistically significant association of *rs143383 *in *GDF5 *core promotor area. Furthermore, the population in our study was in homogeneity and the distribution between the case and control groups was in the Hardy Weinberg's equilibrium. Therefore, our study can represent the significant susceptibility of *GDF5 *in knee osteoarthritis in Thai ethnic. Our findings have emphasized this association that the susceptibility of the polymorphism in *GDF5 *among the Asian population because the magnitude of association is close to Japan and Chinese population. It is possible that Thai population ethnic is believed to be close to Chinese ancestry. Finally, we decide to leave some suggestions in genetic susceptibility study. Firstly, it is important to study the genetic susceptibility in common disease as this might be valuable for further investigation in order to understand the disease at molecular level or even apply to use for disease screening. More important, the association between polymorphism and disease might not similar in different ethics; therefore the genetic susceptibility from different ethics is required and the meta-analysis should be conducted in the adjacent area or similar genetic background. Lastly, other modern technology such as microarray technique should be employed for investigation of genetic susceptibility in osteoarthritis.

## Competing interests

The authors declare that they have no competing interests.

## Authors' contributions

TT conducted the acquisition, analysis and interpretation of data, carried out the molecular genetic studies and drafted the manuscript. TC collected data. SP carried out the genetic study; RFLP. OT participated in the design of the study and performed the statistical analysis. TS and WW participated in its design and coordination and helped to draft the manuscript. All authors read and approved the final manuscript.
